# Relational personhood: the missing link for evaluating clinical impact of brain–computer interfaces

**DOI:** 10.1093/braincomms/fcaf470

**Published:** 2026-01-08

**Authors:** Bouke van Balen, Nick F Ramsey, Mariska J Vansteensel

**Affiliations:** Department of Neurology and Neurosurgery, UMC Utrecht Brain Center, Utrecht 3584 CX, The Netherlands; Human Technology Interaction, Eindhoven University of Technology, Eindhoven 5600 MB, The Netherlands; Ethics and Philosophy of Technology, Delft University of Technology, 2600 GA, The Netherlands; Department of Neurology and Neurosurgery, UMC Utrecht Brain Center, Utrecht 3584 CX, The Netherlands; Donders Institute for Brain, Cognition and Behaviour, Radboud University, Nijmegen 6525 EN, The Netherlands; Department of Neurology and Neurosurgery, UMC Utrecht Brain Center, Utrecht 3584 CX, The Netherlands

**Brain–computer interfaces (BCIs) represent a promising novel neurotechnological solution for people with severe motor and speech impairment to communicate and interact with the world. For clinical adoption, regulatory approval and reimbursement, the clinical outcome assessment of this technology needs to be complemented by the concept of relational personhood.**

Brain–computer interfaces (BCIs) use brain signals to control a computer and represent a highly promising assistive technology to enable people with severe motor and speech impairment to interact with the world. Successful demonstrations of fast and accurate BCI-generated expressions, of unsupervised at-home BCI use, as well as testimonials by BCI research participants, highlight the enormous impact BCI technology can have in their daily lives.^1-3^

With the advent of companies seeking to bring BCIs to market, a key remaining challenge for regulatory approval, reimbursement and clinical implementation of BCIs is the definition of clinical outcome measures that accurately reflect, and can be used to quantify, this impact. Here, we argue that ‘relational personhood’ needs to be integrated in the clinical outcome assessment (COA) of BCIs. This concept captures a fundamental, existential dimension of what BCI technology can contribute to the lives of people with motor and speech impairments and their caregivers, which is not sufficiently covered by currently available clinical outcome measures.

## Why current clinical outcome assessments for BCI fall short

COAs are standardizable assessments that describe or reflect how an individual feels, functions or survives in response to an intervention. Here, we address the subjective, ‘feels’, aspect of the COA of BCIs that enable people to express themselves and interact with the world. In a recent Food and Drug Administration/National Institutes of Health (FDA/NIH) joint workshop on developing COAs for implanted BCIs, a recurring question was how to make sense of the added subjective benefit for BCI users. What does re-enabling a former chess player to play chess again mean besides ‘improved entertainment’? What does it mean for a father and daughter to connect through a BCI besides ‘improved communication’?^1,4^

The discussion on which clinical outcome measures capture the subjective impact experienced by BCI users has so far largely focused on quality of life (QOL), or health-related quality of life (HR-QOL).^5^ Whereas QOL is a general term that expresses the overall wellbeing of an individual, HR-QOL assesses the impact of the individual’s health status (or treatment) on their QOL and is considered a more appropriate concept to evaluate the clinical impact of medical products than QOL. Yet, the clinical impact of a BCI may not be sufficiently captured by QOL or HR-QOL for two reasons. First, many currently available tools to assess HR-QOL are generic, include questions related to physical functioning that can be perceived as insensitive by people with severe motor impairment and focus insufficiently on the social consequences of the impairment, which matter more to patients than the physical disability per se.^5^ Second, it may prove difficult to demonstrate a meaningful improvement in (HR-)QOL as a result of BCI use, given that, at least a subportion of, people with severe motor and speech impairments already report a high QOL, or relatively high values in the emotional domains of HR-QOL scales, without having access to BCI technology.^6^ Indeed, it is well described that people with chronic conditions tend to adjust to their situation over time and develop a ‘response shift’: by adapting their expectations and priorities, they may experience a high (HR-)QOL despite a decline in health.^7^

High baseline (HR-)QOL values may lead to the assumption or conclusion that BCI technology does not contribute to the subjective experiences of users. However, this is at odds with testimonies from research participants in clinical trials, which suggest that the clinical impact of BCI technology on how people feel can be substantial. Conveying the essence of this impact is the question raised by one of the participants of the FDA/NIH workshop: ‘how do we capture that reconnection between father and daughter after using an implanted BCI technology on Day 2 of use?’^4^

## Relational personhood as the missing link

This question is valid. Indeed, something fundamental seems to be happening to people using a BCI to express themselves as well as to their caregivers. We argue that this ‘something’ can be captured by the concept of relational personhood, stemming from philosophy.^8^ Relational personhood expresses whether and to what extent people are, and feel perceived as, full persons by each other.

Our bodies allow us to express ourselves, which not only enables us to be perceived as persons with rich inner worlds by others but also to acknowledge the personhood of the people around us. People’s ability to express themselves is thereby an enabling factor for relational personhood. People living with severe paralysis often experience being seen and treated as objects instead of persons.^9^ At the same time, the affected individual is less able of acknowledging their caregivers and loved ones as full persons. As communication becomes more challenging, it focuses increasingly on basic (care) needs, and interactions about the inner worlds of both parties diminish. The painstaking aspect of this experience is that people with motor and speech impairments have no less rich inner worlds than others. Their impairment does not make them less of a person, and yet they are seen and treated less as full persons by others and are less capable of treating other people as such.

BCI technology enables people with motor and speech impairments and the people around them to regain access to each other’s inner worlds. Being able to play chess again if it was a former hobby means something more than entertainment. It means being able to express a part of your personality to the world and to be able to interact with, and challenge, other players. Father and daughter reconnecting through a BCI means more than the disabled father’s ability to have better and more accurate communication. What it means from a subjective perspective is two loved ones who become able to perceive each other to a greater extent as the persons they are to each other. It is a reconnection between father and daughter in the truest sense of the word—father’s personhood becomes more perceivable for his daughter, and he is able to express to his daughter how he sees her as a person. He does not merely regain the ability to communicate, but they in essence re-establish their father–daughter relationship.

Relational personhood is a multi-faceted concept that encompasses phenomena such as identity, autonomy, dignity, relationality and the social nature of human beings. All of these phenomena are related to fundamental human rights and are at stake when self-expression is impaired or lost completely. As such, we believe it is necessary to include relational personhood as a complement patient-reported outcome measure to existing clinical outcome measures for BCIs. Relational personhood has been raised before as a potential target for BCIs,^10^ but has never been clearly operationalized and, to our knowledge, has so far been largely absent from discussions on COAs for BCIs.

## Development of a communication and relational personhood questionnaire

Several (HR-)QOL questionnaires lightly touch upon topics that are associated with relational personhood. For example, the SF-36 includes a question about social activities, and the ALSSQOL-R refers to emotional intimacy and the satisfactoriness of relationships. Yet, validated instruments or questionnaires to comprehensively quantify the impact of motor and speech impairment and of assistive technology on relational personhood seem to not exist, and we here argue that such a tool needs to be created for the COA of BCIs. For a complete impact assessment, we foresee several modules, where in addition to a general question module, more specific modules address function-specific experiences. The tool needs to include questions for both primary users and their loved ones/caregivers, since the experience of personhood is relational and BCI use affects both parties. The questions should cover the multi-faceted phenomena associated with relational personhood, such as identity (e.g. to what extent does the BCI help you to express, and be seen for, who you are?), autonomy (e.g. to what extent does the BCI help you to be, and feel, in control of your daily life?), dignity (e.g. to what extent does the BCI make you feel respected by your loved one/others?) and relationality (e.g. to what extent does the BCI help you to establish and maintain meaningful relationships, or to what extent does the BCI help you to be the parent/spouse/friend you want to be?). The tool needs to be developed using input from the various types of BCI stakeholders and validated according to established procedures.

## Conclusion

In conclusion, we here call upon the BCI community and stakeholders to contribute to the development and validation of an innovative communication and relational personhood questionnaire (CRPQ) that captures the fundamental, existential impact of BCIs and merits broad support. This tool and our perspective are applicable to other types of assistive and augmentative communication technologies that support self-expression and communication for people with disabilities. Once developed and validated, the CRPQ will complement the more conventional clinical outcome measures, such as (HR-)QOL, to gain a complete understanding of the clinical benefit of BCIs, eventually supporting regulatory approval, clinical adoption and reimbursement of BCIs.

## References

[1] Card NS, Wairagkar M, Iacobacci C, et al An accurate and rapidly calibrating speech neuroprosthesis. N Engl J Med. 2024;391(7):609–618. 39141853 10.1056/NEJMoa2314132 PMC11328962

[2] Metzger SL, Littlejohn KT, Silva AB, et al A high-performance neuroprosthesis for speech decoding and avatar control. Nature. 2023;620(7976):1037–1046. 37612505 10.1038/s41586-023-06443-4 PMC10826467

[3] Vansteensel MJ, Leinders S, Branco MP, et al Longevity of a brain–computer interface for amyotrophic lateral sclerosis. N Engl J Med. 2024;391(7):619–626. 39141854 10.1056/NEJMoa2314598 PMC11395392

[4] Wu G, Keszler M, Slocomb J, Dean H. FDA/NIH joint workshop—developing implanted brain-computer interface clinical outcome assessments to demonstrate benefit. Published online 2024. Accessed 13 October 2025. https://www.fda.gov/media/182567/download.

[5] Fry A, Chan HW, Harel NY, Spielman LA, Escalon MX, Putrino DF. Evaluating the clinical benefit of brain-computer interfaces for control of a personal computer. J Neural Eng. 2022;19(2):021001. 10.1088/1741-2552/ac60ca 35325875

[6] Prell T, Gaur N, Steinbach R, Witte OW, Grosskreutz J. Modelling disease course in amyotrophic lateral sclerosis: Pseudo-longitudinal insights from cross-sectional health-related quality of life data. Health Qual Life Outcomes. 2020;18(1):117. 32357946 10.1186/s12955-020-01372-6 PMC7195704

[7] Sprangers MA, Schwartz CE. Integrating response shift into health-related quality of life research: A theoretical model. Soc Sci Med. 1999;48(11):1507–1515. 10400253 10.1016/s0277-9536(99)00045-3

[8] Van Grunsven J, Van Balen B, Bollen C. Three embodied dimensions of communication. In: De Boer B, Zwier J, eds. Phenomenology and the philosophy of technology. Open Book Publishers; 2024:241–266.

[9] Nizzi MC, Blandin V, Demertzi A. Attitudes towards personhood in the locked-in syndrome: From third- to first-person perspective and to interpersonal significance. Neuroethics. 2020;13(2):193–201.

[10] Kübler A . The history of BCI: From a vision for the future to real support for personhood in people with locked-in syndrome. Neuroethics. 2020;13(2):163–180.

